# Iguratimod Restrains Circulating Follicular Helper T Cell Function by Inhibiting Glucose Metabolism *via* Hif1α-HK2 Axis in Rheumatoid Arthritis

**DOI:** 10.3389/fimmu.2022.757616

**Published:** 2022-06-01

**Authors:** Ziran Bai, Zhimin Lu, Rui Liu, Yawei Tang, Xiaokang Ye, Minli Jin, Guan Wang, Xia Li

**Affiliations:** ^1^ Department of Immunology, College of Basic Medical Science, Dalian Medical University, Dalian, China; ^2^ Department of Rheumatology, Affiliated Hospital of Nantong University, Nantong, China; ^3^ Department of Rheumatology, The First Affiliated Hospital of Nanjing Medical University, Nanjing, China

**Keywords:** rheumatoid arthritis, circulating follicular helper T cells, iguratimod, glucose metabolism, Hif1α-HK2 axis

## Abstract

Iguratimod (IGU) is a novel disease modified anti-rheumatic drug, which has been found to act directly on B cells for inhibiting the production of antibodies in rheumatoid arthritis (RA) patients. Follicular helper T (Tfh) cells, a key T cell subsets in supporting B cell differentiation and antibody production, have been shown to play critical roles in RA. However, whether IGU can inhibit RA Tfh cells which further restrains B cell function remains unclear. Here, we aimed to explore the roles of IGU in regulating RA circulating Tfh (cTfh) cell function and investigate the potential mechanism associated with cell glucose metabolism. In our study, we found that IGU could act on RA-CD4^+^ T cells to reduce T cell-dependent antibody production. IGU decreased the percentage of RA cTfh cells and the expression of Tfh cell-related molecules and cytokines which were involved in B cell functions. Importantly, our data showed that IGU significantly restrained the cTfh cell function by inhibiting glucose metabolism, which relied on Hif1α-HK2 axis. In summary, we clarified a new target and mechanism of IGU by restraining RA cTfh cell function *via* inhibiting Hif1α-HK2-glucose metabolism axis. Our study demonstrates the potential application of IGU in the treatment of diseases related to abnormal metabolism and function of Tfh cells.

## Introduction

Rheumatoid arthritis (RA) is a chronic inflammatory condition characterized by articular synovitis, ultimately leading to functional impairment and disability (1). Although the pathogenesis of RA remains unclear, numerous studies have demonstrated that the autoantibodies produced by B cells play a pivotal role in the pathogenetic processes of RA (2). The proliferation and differentiation of antigen-primed B cells essentially rely on the helper function of CD4^+^ T cells. Follicular helper T (Tfh) cells are identified as a subset of CD4^+^ T cells that specialize in helping B cells for the formation and maintenance of the germinal center (GC), the production of antibodies, and long-lived plasma cells (1, 3). In particular, the differentiation and function of Tfh cells were involved in a range of autoimmune diseases, including RA (1).

Iguratimod (IGU or T-614) is a novel synthetic small molecule disease modified anti-rheumatic drug (DMARD), which is approved only in Japan and China (4). A series of clinical studies on IGU in Japan and China confirmed that IGU could be used as a new option for RA treatment. IGU has good efficacy and tolerance as an additional treatment for RA patients with inadequate response to methotrexate (MTX) and biological DMARDs (5). Pharmacological studies have shown that IGU can reduce the production of immunoglobulin (Ig) by acting on B cells and can also accelerate bone formation by inhibiting the activation of osteoclasts and promoting osteoblast differentiation (4, 6, 7). However, the role of IGU in regulating the specific biological properties of Tfh cells in RA patients and its mechanism remains unclear.

Increasing evidence indicates that cellular energy metabolism directs the survival, proliferation, and immune responses of T cells (8). After recognizing the specific antigen, T cells expand clonally, enter the inflammatory site and obtain effector function. These processes have significant bioenergetic and biosynthetic demands, which are met by dynamic changes in T-cell metabolism, specifically increases in glucose uptake and metabolism (8). Hexokinases (HKs) catalyze the first committed step in glucose metabolism. By catalyzing the phosphorylation of glucose to glucose 6-phosphate (G6P), HKs promote and sustain a concentration gradient that facilitates glucose entry into cells and the initiation of all major pathways of glucose utilization (9). The specific HK2 inhibitor can significantly decrease the arthritis scores and the histological scores in an autoimmune model of RA (10). A study has also shown that inhibiting glycolysis can uniquely target pathogenic autoreactive Tfh cells (11). Several molecular signaling pathways and/or molecules have been identified, which are critical and required for T cell metabolic programming and development. Recent studies have demonstrated that the mammalian/mechanistic target of rapamycin (mTOR) signaling plays a critical role in regulating glucose uptake and energy balance (12). Hypoxia-inducible factor 1α (Hif1α) also serves as a key transcription factor that performs essential functions in the regulation of cellular metabolism, especially in the regulation of HK2 expression (13). Further study on the mechanism of glucose metabolic programming in T cells, especially in Tfh cells, will provide clues for new metabolic therapy for autoimmune diseases.

Chemical immunosuppressive drugs including mTOR inhibitors (sirolimus and everolimus), calcineurin inhibitors (tacrolimus and cyclosporine), and purine and pyrimidine synthesis inhibitors (6-mercaptopurine, mycophenolic acid, and methotrexate) are widely prescribed for the treatment of autoimmune and inflammatory diseases and for controlling alloimmunity in interfering with the signals that activate and allow T cells to proliferate. Emerging evidence indicates that these drugs also target T-cell metabolism and metabolic checkpoints, contributing to their immunosuppressive effects (14, 15). In consideration of the key role of glucose metabolism in T cell functions, we hypothesized whether IGU could regulate the functions of the Tfh cells by reprogramming glucose metabolism.

In our study, we aimed to explore the roles of IGU in regulating RA circulating Tfh (cTfh) cell function and investigate the potential mechanism associated with cell glucose metabolism.

## Materials and Methods

### Human Blood Samples

Peripheral blood from RA patients was obtained from the Department of Rheumatology and Immunology of the Second Affiliated Hospital of Dalian Medical University in China. All RA patients in this study fulfilled the American College of Rheumatology (ACR) 1987 revised criteria. Age and sex-matched health controls (HC) were obtained from the Medical Examination Center of the Second Hospital of Dalian Medical University. The study has been approved by the ethics committee of the Dalian Medical University (2018–061), and informed consent was obtained from all subjects. The median age of RA patients was 52.40 ± 11.18 years and HC was 40.80 ± 12.75 years.

### Chemical

Iguratimod (IGU) was provided by Simcere Pharmaceutical (Nanjing, China).

### Cell Isolation, Purification, and Activation

Peripheral blood mononuclear cells (PBMCs) were purified from peripheral blood using Ficoll-Paque plus. Human CD4^+^ T and CD19^+^ B cells were purified from PBMCs by Miltenyi Cell Isolation Kit according to the manufacturer’s protocols. The purified CD4^+^ T cells and PBMCs were activated with anti-CD3 and anti-CD28 antibodies (eBioscience) in RPMI-1640 containing 10% FBS and treated with vehicle (DMSO) or IGU (0-200 μg/ml) for 24-72 hours. In several experiments, 2-DG (5 mM) (Solarbio), Rapamycin (100 nM) (Sigma-Aldrich), Echinomycin (5 nM) (Abcam), or Adaptaquin (5 μM) (R&D Systems) were administrated to the cell culture.

### T Cell-B Cell Coculture

CD4^+^ T cells were purified from PBMCs of RA patients using Human CD4^+^ T Cell Isolation Kit (Miltenyi) and then cultured with plate-coated anti-CD3 antibody (5 μg/ml) and anti-CD28 antibody (2 μg/ml) plus IGU or DMSO in RPMI-1640 containing 10% FBS for 3 days. CD19^+^ B cells were purified from PBMCs of healthy donors using Human CD19^+^ B cell Isolation Kit (Miltenyi). In a 96-well round-bottom plate, CD19^+^ B cells were co-cultured with RA-CD4^+^ T cells which were treated with or without IGU at a ratio of 5:1. A total of 2 μg/ml anti-CD3/CD28, 0.5 μg/ml anti-CD40 antibody (R&D Systems), 0.1 μM CpG (Miltenyi) were added to RPMI 1640 complete medium, which was then added to the cells at 200 μl per well. After 7 days, the supernatant was separated from the cells *via* centrifugation. Both cells and cell culture supernatant were collected for further experiments.

### Quantitative Polymerase Chain Reaction (qPCR)

RA-CD4^+^ T cells stimulated by plate-coated anti-CD3 antibody (5 μg/ml) and anti-CD28 antibody (2 μg/ml) with or without IGU for 24 hours were collected and washed with ice-cold PBS twice. Total RNA was extracted from RA-CD4^+^ T cells using AG RNAex Pro Reagent (AG21102, Accurate Biology, Hunan, Co., Ltd), and cDNA was transcribed using 5×All-In-One RT MasterMix (Abm), both according to manufacturers’ instructions. Real-time PCR was carried out with the SYBR Green Premix Pro Taq HS qPCR Kit (AG11701, Accurate Biology, Hunan, Co., Ltd) on the CFX96 Real-Time PCR Detection System (Bio-Rad). The mRNA levels in each sample were normalized to the relative quantity of β-actin gene expression and relative gene expression was determined using the standard 2^-ΔΔCT^ method. The specific primers used are listed in **Supplemental Table 1**.

### Glucose Uptake Assay

Glucose uptake was measured following 20 min incubation with a fluorescent D-glucose analog 2-[N-(7-nitrobenz-2-oxa-1,3-diazol-4-yl) amino]-2-deoxy-D-glucose (2-NBDG) (Sigma-Aldrich). PBMCs from RA patients and healthy donors were resuspended in glucose-free RPMI 1640 medium (Gibco) for 30 min, and then 50 μM of 2-NBDG was added. In addition, purified RA-CD4^+^ T cells were treated with IGU or DMSO for 24 hours, then cultured in glucose-free RPMI 1640 medium for 30 min, and followed by adding of 2-NBDG (50 μM). After 20 min, cells were placed on ice and washed twice with ice-cold PBS. Cells were measured immediately by flow cytometry after CD4 staining.

### Glucose and L-Lactate Assays

Purified RA-CD4^+^ T cells were treated with IGU and DMSO for 24 hours. Glucose concentrations in the cell culture supernatants were measured by Glucose Assay Kits (Sigma-Aldrich). L-lactate concentrations in cell culture supernatants from RA-CD4^+^ T cells were determined using the CheKine Lactate Assay Kit (Abbkine). The glucose and L-lactate concentrations were determined according to the manufacturer’s protocols. Glucose uptake = glucose concentration in complete medium - glucose concentration in cell culture supernatants.

### Metabolic Profiling

Real-time measurements of extracellular acidification rate (ECAR) and oxygen consumption rate (OCR) were performed with a Seahorse XF24 Extracellular Flux Analyser (Agilent). To detect the regulation of IGU on CD4^+^ T cell metabolism, RA-CD4^+^ T cells were pre-activated by anti-CD3 antibody and anti-CD28 antibody for 24 hours and then treated with DMSO or IGU 1 hour before the experiment. Collecting and seeding the cells in XF Assay medium in a 24-well microplate pre-coated with 0.01% Poly-L-lysine (Solarbio). Cellular oxidative phosphorylation was determined by Seahorse XF Cell Mito Stress test kit with three injections: 1 μM oligomycin, 1 μM FCCP, and 1 μM rotenone. Glycolysis associated parameters were determined by Seahorse XF Glycolysis Stress test kit with three injections: 10 mM glucose, 1 μM oligomycin, and 50 mM 2-DG.

### Cell Proliferation Assay

Cell proliferation assays were performed using the Carboxyfluorescein succinimidyl amino ester (CFSE) Cell Labeling Assay (eBioscience). PBMCs isolated from healthy donors and RA patients were washed two times with PBS and resuspended at pre-warmed PBS. CFSE was added at a final concentration of 1.5 μM to label cells. The CFSE labeled cells were stimulated by anti-CD3/CD28 antibodies (2 μg/ml) in the presence of DMSO or IGU for 3 or 5 days. Finally, the cells were washed with PBS twice and measured by flow cytometry after CD4 staining.

### Cell Apoptosis Analysis

Cell apoptosis was analyzed by using the Propidium Iodide (PI) and Annexin V-FITC Apoptosis Test Kit (eBioscience). PBMCs isolated from healthy donors or RA patients were stimulated by anti-CD3/CD28 antibodies (2 μg/ml) with or without IGU for 24 hours. Then, the cells were harvested and washed with cold PBS twice. Next, the cells were resuspended in 100 μl binding buffer to stain CD4 and Annexin V-FITC in the dark. After 20 min, the cells were rewashed, and PI was added. The early apoptotic cells were detected by flow cytometry.

### Flow Cytometry Analysis

Cell surface staining was performed using BD Biosciences or eBioscience reagents. The dead cells were excluded from the analysis by fixable viability dye (FVD) (eBioscience) before labeling the surface antibody except for cell apoptosis analysis. For intracellular cytokine and phosphorylation mTOR (p-mTOR) staining, cells were permeabilized using the Cytofix/Cytoperm Intracellular Staining Kit (BD Biosciences) and stained with cytokine-specific mAbs. For Bcl-6, Hif1α, and Foxp3 staining, cells were fixed and permeabilized using the Transcription Factor Fixation/Permeabilization Buffer Set (eBioscience). For HK2 staining, the cells were fixed in 4% paraformaldehyde and permeabilized in 0.5% Triton X-100 before antibody staining. For LDH staining, the cells were fixed in 4% paraformaldehyde and permeabilized in 90% methanol before antibody staining. The flow antibodies are listed in **Supplemental Table 2**. All stained cells were analyzed on the Flow Cytometer (NovoCyte 2040R) and data were analyzed with NovoExpress software.

### Enzyme-Linked Immunosorbent Assay (ELISA)

After the T-B cell was co-cultured for 7 days, the supernatant was separated from the cells *via* centrifugation. The concentrations of IgM and IgG in the supernatant were measured using the Human IgM ELISA Kit and the Human IgG ELISA Kit (SenBeiJia Biological Technology, China) according to their instructions. The plates were read at 450 nm by a microplate reader (Thermo).

### Western Blotting

For western blot analyses, proteins were extracted by lysing anti-CD3/CD28 activated RA-CD4^+^ T cells in RIPA buffer. Equal amounts of protein in each sample were analyzed by SDS-PAGE and electrophoretically transferred to nitrocellulose filter (NC) membranes. The membranes were incubated with antibodies including HK2 (anti-rabbit, Cell Signaling Technology), LDHA (anti-rabbit, Cell Signaling Technology), β-actin (anti-rabbit, Cell Signaling Technology) at 4 °C overnight, then incubated with a fluorescent secondary antibody (Abclonal) for 1.5 hours at room temperature. The results were detected by Odyssey CLx Infrared Scanner (Odyssey CLx). The expression levels of HK2 and LDH were normalized to β-actin and adjusted to the levels in the control group (served as 1).

### Statistical Analysis

Statistical analysis was performed with GraphPad Prism 9 software. Unless indicated otherwise, data are expressed as mean ± standard error of mean (SEM). Statistical differences were analyzed by unpaired Student t-test, paired Student’s t-test, Mann-Whitney test, and one-way ANOVA. The *P*-values < 0.05 were considered significant. Asterisks mark the significant differences between different groups (*, *P* < 0.05; **, *P* < 0.01; ***, *P* < 0.001 and ****, *P* < 0.0001).

## Results

### IGU Inhibits T Cell-Dependent Antibody Production

It is generally believed that IGU plays an important immunomodulatory role by inhibiting the production of immunoglobulins on B cells (16, 17). However, B cells require interaction with helper CD4^+^ T cells to become activated (18). The interactions of CD4^+^ T cells and B cells are fundamental for the generation of antibody responses, as well as for the development of harmful autoimmune diseases (19, 20). We wondered whether IGU might regulate T-B interactions. We first screened the optimal concentration of IGU in CD4^+^ T cells, detecting the proliferation and apoptosis of HC CD4^+^ T cells that were treated with different concentrations of IGU (0, 10, 50, 100, 150, 200 μg/ml). The gating strategy of CD4^+^ T cells was shown in **Figure S1A**. The results showed that 100 μg/ml of IGU could significantly inhibit the proliferation of CD4^+^ T cells (**Figure S1B**) but had no obvious effect on their apoptosis (**Figure S1C**). Therefore, we selected 100 μg/ml IGU for the following studies.

**Figure 1:**
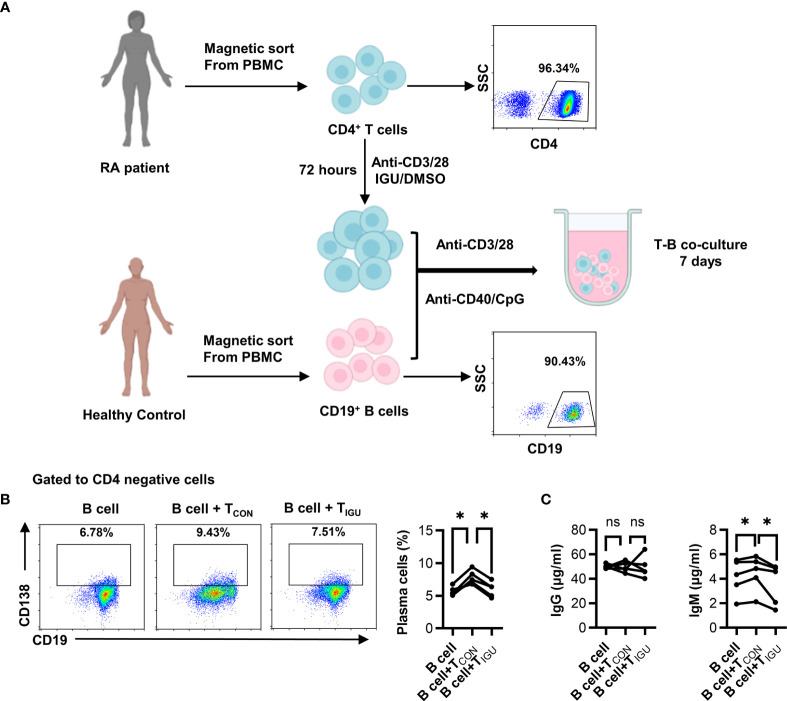
IGU inhibits T cell-dependent antibody production. **(A)** Purified RA-CD4^+^ T cells were cultured in the presence or absence of IGU for 72 hours, then co-cultured with CD19^+^ B cells from healthy donors for 7 days. The purity of CD4^+^ T cells and CD19^+^ B cells were shown. **(B)** The percentage of plasma cells (CD138^+^) was detected by flow cytometry (n = 5). **(C)** The production of IgG and IgM in the supernatant was detected by ELISA (n = 5). Symbols represent individual subjects. ns, no significance; *P < 0.05.

To examine whether IGU impinges on T-B interactions, we conducted *in vitro* conjugation assay. We first treated RA-CD4^+^ T cells with IGU or the equal volume of DMSO for 3 days, then co-cultured with CD19^+^ B cells for 7 days. The purity of CD4^+^ T cells was higher than 95% and the purity of CD19^+^ B cells was higher than 90% (**Figure 1A**). As compared with the B cell-control RA-CD4^+^ T cell co-culture group (B + T_CON_), the percentage of CD138^+^ plasma cells decreased in the B cell + IGU treated RA-CD4^+^ T cell co-culture group (B + T_IGU_) (**Figure 1B**). Simultaneously, we also found that although there was no significant difference in IgG level in the supernatant of the co-culture system, IgM level decreased when CD19^+^ B co-cultured with IGU treated RA-CD4^+^ T cells (**Figure 1C**). These results hint that IGU can inhibit T cell-dependent antibody production.

### IGU Inhibits RA-CD4^+^ T Cell Proliferation and Activation

**Figure 2:**
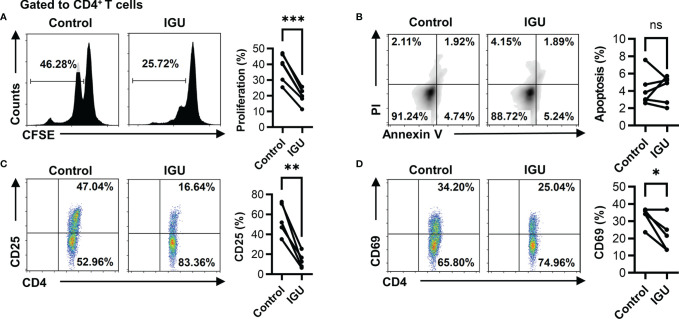
IGU inhibits RA-CD4^+^ T cell proliferation and activation. The PBMCs from RA patients were cultured in the presence of anti-CD3/CD28 antibody (2 μg/ml) in the presence of vehicle (DMSO) or IGU for 5 days. **(A)** The proliferation of RA-CD4^+^ T cells was detected by the CFSE labeling method (n = 6). **(B)** The apoptosis of RA-CD4^+^ T cells was detected after being cultured for 24 hours (n = 6). Propidium iodide (PI) and Annexin V (AV) staining were determined by flow cytometry. The data of early apoptotic cells (PI^−^AV^+^) were shown. **(C, D)** RA-PBMCs were cultured in the presence or absence of IGU for 72 hours. Flow cytometry detected the expression of activation markers (CD25 and CD69) on CD4^+^ T cells (n = 5). Symbols represent individual subjects. ns, no significance; *P < 0.05; **P < 0.01; ***P < 0.001.

Next, we want to explore what function of T cells is affected by IGU. Firstly, we examined whether IGU regulated RA-CD4^+^ T cell proliferation and apoptosis. We found that IGU inhibited the proliferation of RA-CD4^+^ T cells (**Figure 2A**) but had no significant effect on their apoptosis (**Figure 2B**). We then tested the effects of IGU on the activation of RA-CD4^+^ T cells. We found that activation markers, including CD25 and CD69, decreased significantly on day 3 in the presence of IGU (**Figures 2C** **, D**). Collectively, these results indicate that IGU inhibits RA-CD4^+^ T cells proliferation and activation.

### IGU Inhibits the cTfh Cell Function of RA Patients

**Figure 3:**
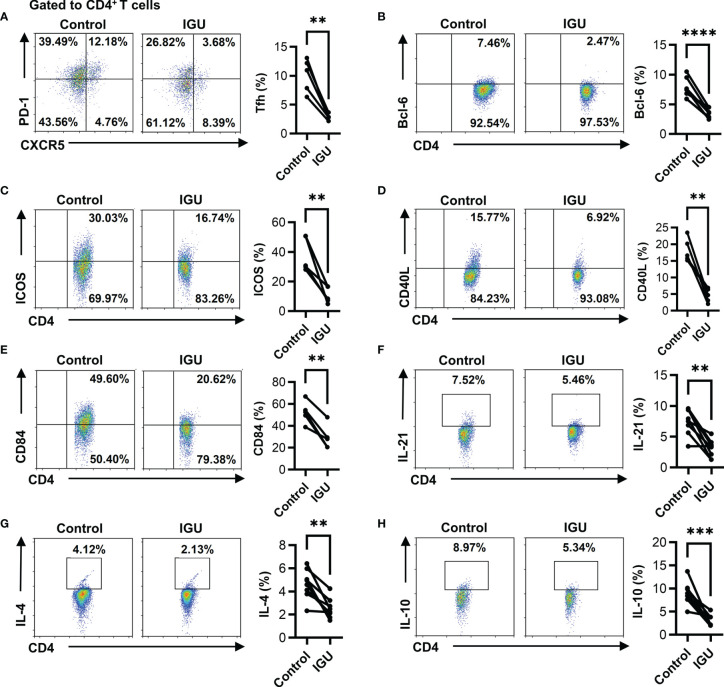
IGU inhibits the cTfh cell function of RA patients. RA PBMCs activated by anti-CD3/CD28 antibody (2 μg/ml) were cultured with IGU or DMSO for 72 hours. **(A)** The percentage of Tfh (CD4^+^CXCR5^+^PD-1^+^ T) cells was measured by flow cytometry (n = 5). **(B)** The percentage of CD4^+^Bcl-6^+^ T cells was measured by flow cytometry (n = 8). **(C–E)** The expression of ICOS, CD40L, and CD84 on CD4^+^ T cells were detected by flow cytometry (n = 5). **(F–H)** The secreted cytokines of CD4^+^ T cells, including IL-21, IL-4, and IL-10, were measured by flow cytometry (n = 8). Symbols represent individual subjects. **P < 0.01; ***P < 0.001; ****P < 0.0001.

The proportion of cTfh (CD4^+^CXCR5^+^PD-1^+^ T) cells in RA patients was higher than that in HC (**Figure S2A**). Next, we evaluated the percentages of cTfh cells after IGU treatment. As shown in **Figure 3A**, the percentages of cTfh cells significantly decreased in the presence of IGU. Tfh cells are regulated by a complicated network of transcription factors, including B cell lymphoma 6 protein (Bcl-6) and basic leucine zipper ATF-like transcription factor (BATF) (3). We purified CD4^+^ T cells from the PBMCs of RA patients and treated them with or without IGU. We observed that the expression of Bcl-6 and BATF significantly decreased at the mRNA level after IGU treatment (**Figure S2B**). Flow cytometry results showed that the percentages of CD4^+^Bcl-6^+^ T cells were also down-regulated after IGU treatment (**Figure 3B**). Tfh cells provide help to cognate B cells *via* their expression of CD40 ligand (CD40L), interleukin (IL)-21, IL-4, and other characteristic markers (21). Thus, we examined the surface molecules and cytokines which are related to Tfh cell function. We found that the expression of ICOS, CD40L, and CD84 (**Figures 3C** **–E**) on RA-CD4^+^ T cells significantly decreased, and we found that IL-21, IL-4, and IL-10 secreted by CD4^+^ T cells also reduced (**Figures 3F** **–H**). These results indicate that IGU can inhibit the RA-cTfh cell function.

In addition, peripheral helper T (Tph), Th1, Th17, and Treg cells are also involved in the pathogenesis of RA by secreting different cytokines (22, 23). As shown in **Figures S2C–F**, we found that IGU could also inhibit the percentage of Tph (CD4^+^PD-1^+^CXCR5^-^ T) cells, CD4^+^IFN-γ^+^ T cells, CD4^+^IL-17A^+^ T cells, but had no significant effect on Treg (CD4^+^Foxp3^+^ T) cells.

### IGU Inhibits Glucose Metabolism in RA-CD4^+^ T Cells

Cellular glucose metabolism controlling the functions of immune cells has greatly evolved in the past few years. Moreover, inhibition of glycolysis can significantly decrease the severity of joint inflammation in a model of RA (10). We further consider whether IGU can regulate the glucose metabolism in RA-CD4^+^ T cells. Firstly, we characterized the glucose metabolism ability of RA and HC CD4^+^ T cells. We found no significant difference in glucose uptake ability in RA and HC groups (**Figure S3A**). However, our results demonstrated that the expression of key enzymes such as glucose transporter type 1 (GLUT1) and HK2 in RA-CD4^+^ T cells were higher than in HC-CD4^+^ T cells (**Figures S3B, C**), although there was no significant difference in lactate dehydrogenase (LDH) expression (**Figure S3D**). These phenomena suggest that RA-CD4^+^ T cells may have more active glucose metabolism than HC.

**Figure 4:**
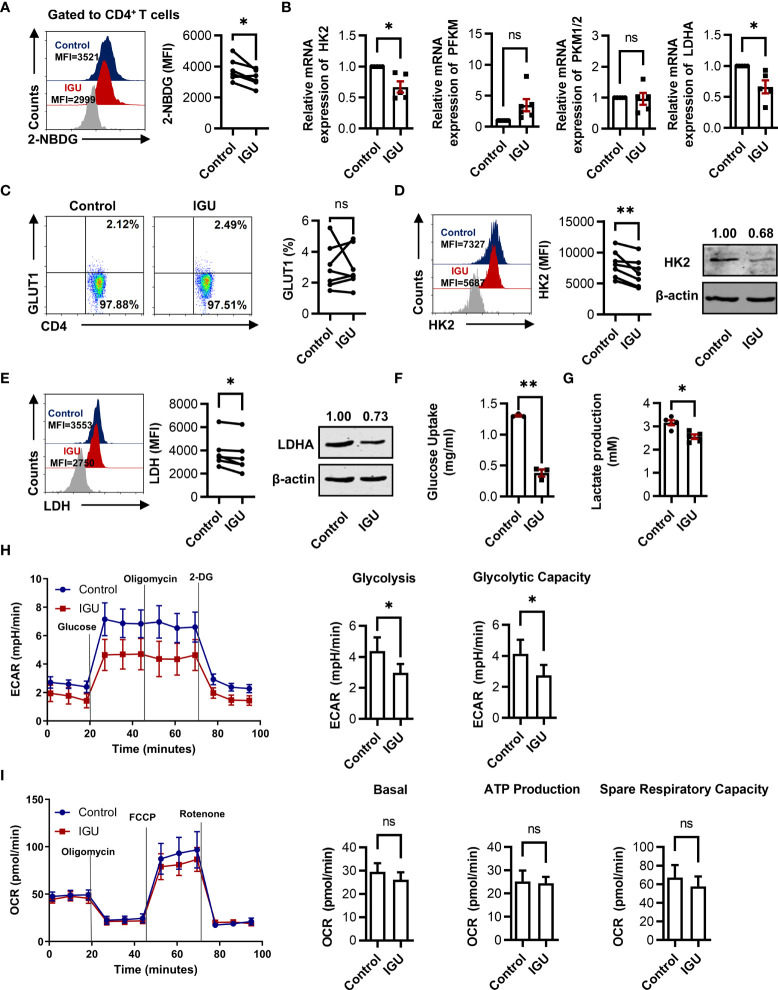
IGU inhibits glucose metabolism in RA-CD4^+^ T cells. **(A)** RA-PBMCs were cultured in the presence of DMSO or IGU for 24 hours, then the glucose uptake was determined by the 2-NBDG method (n = 6). **(B)** Purified RA-CD4^+^ T cells were cultured in the presence of IGU or DMSO for 24 hours, and relative mRNA expression levels of HK2, PFKM, PKM1/2, and LDHA were determined by qPCR. Expression levels of each gene were normalized to β-actin expression level and adjusted to the levels in the control group (served as 1). **(C-E)** GLUT1 (n = 7), HK2 (n = 8), and LDH (n = 6) in RA-CD4^+^ T cells were measured by flow cytometry and western blotting. **(F, G)** Purified RA-CD4^+^ T cells were cultured in the presence of IGU or DMSO for 24 hours. Glucose uptake (n = 3) and lactate production (n = 5) in the culture supernatants were determined by the glucose and lactate assay kit. **(H, I)** RA-CD4^+^ T cells were activated by anti-CD3/CD28 for 24 hours, then treated with DMSO or IGU for 1 hour. ECAR (n = 5) and OCR (n = 5) for each group were measured in real-time under basal conditions and after addition of the indicated reagents. The data of glycolysis, glycolytic capacity, basal OCR, ATP production, and spare respiratory capacity were shown. MFI: mean fluorescence intensity. Symbols represent individual subjects. ns, no significance; *P < 0.05; **P<0.01.

To explore whether IGU can also regulate the glucose metabolism of RA-CD4^+^ T cells, we determined the potential suppression of the glucose uptake ability in RA-CD4^+^ T cells during IGU-mediated function inhibition. We found that IGU treatment markedly inhibited the glucose uptake ability in RA-CD4^+^ T cells (**Figure 4A**). We also determined the key enzymes of the glycolytic pathway, including HK2, muscle-type phosphofructokinase (PFKM), pyruvate kinase M1/2 (PKM1/2), and lactate dehydrogenase A (LDHA) by using qPCR. The results showed that the mRNA expression of HK2 and LDHA decreased in the IGU treated group, while no effect was observed in PFKM or PKM1/2 (**Figure 4B**). As some studies have reported that RA T cells are characterized by the increased availability of the pentose phosphate pathway (24), we next examined two key enzymes that regulate the pentose phosphate pathway, 6-phosphofructo-2-kinase/fructose-2,6-biphosphatase 3 (PFKFB3) and glucose-6-phosphate dehydrogenase (G6PD). However, IGU did not affect these two enzymes (**Figure S3E**). Furthermore, we analyzed the protein levels of GLUT1, HK2, and LDH (**Figures 4C** **–E**). Consistent with the mRNA levels, the protein levels of HK2 and LDH were significantly down-regulated after IGU treatment, while the expression of GLUT1 had no significant change. Concurrently, we also detected the supernatant of CD4^+^ T cells treated with IGU or DMSO and found that the glucose uptake (**Figure 4F**) and lactate production (**Figure 4G**) of CD4^+^ T cells were significantly inhibited in the presence of IGU. To further verify the direct effect of IGU on CD4^+^ T cell glucose metabolism, we measured the real-time bioenergetic profiles: ECAR, an indicator of glycolysis, and OCR, an indicator of OXPHOS (**Figures 4H** **, I**). Notably, compared with the control groups, IGU treated CD4^+^ T cells showed lower glycolysis and glycolytic capacity. However, there was no significant difference in basal OCR, ATP production, and spare respiratory capacity. Collectively, these results indicate that IGU inhibits glucose metabolism in RA-CD4^+^ T cells.

### IGU Restrains the Function of cTfh Cells by Inhibiting HK2

**Figure 5:**
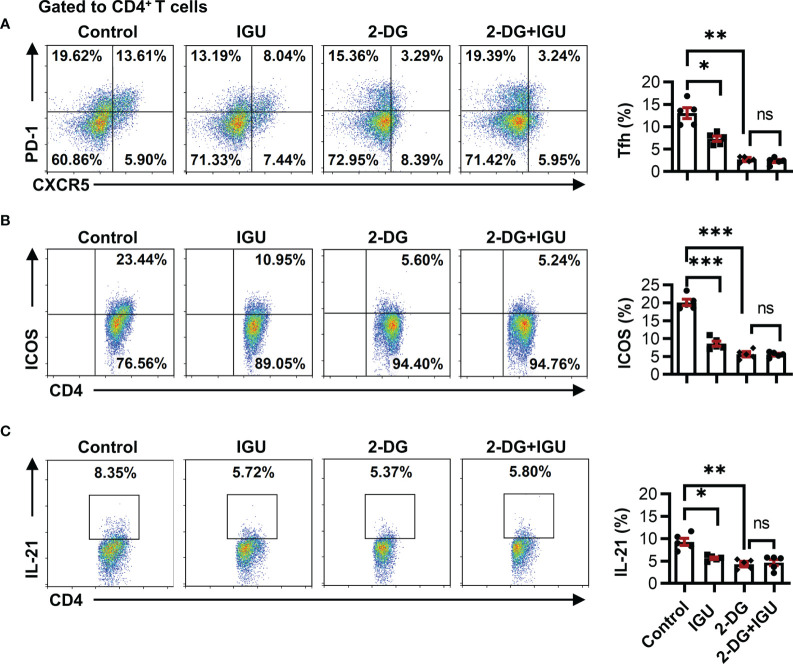
IGU restrains the function of cTfh cells by inhibiting HK2. RA-PBMCs were activated by anti-CD3/CD28 antibody (2 μg/ml) and pretreated with HK2 inhibitor 2-DG or not for 4 hours, and then treated with IGU or DMSO for 3 days. **(A–C)** The frequencies of Tfh cells **(A)**, ICOS^+^CD4^+^ T cells **(B)**, and IL-21 producing CD4^+^ T cells **(C)** were determined by flow cytometry (n = 5). ns, no significance; *P < 0.05; **P<0.01; ***P < 0.001.

A recent study has shown that high glucose utilization is a unique requirement of autoreactive Tfh cells and has implied that inhibiting glycolysis can uniquely target pathogenic autoreactive Tfh cells while preserving protective immunity against pathogens (11). Both pentose phosphate pathway and glycolysis will work through HK2, which is the first-rate limiting enzyme of glucose metabolism. We determined whether the inhibitory effect of IGU on RA-CD4^+^ T cells was dependent on the inhibition of HK2 by using a specific pharmacological inhibitor, 2-DG, which had no effect on cell apoptosis (**Figure S4**). Consistent with the previous reports (25), the frequencies of cTfh cells, the activated molecules ICOS on CD4^+^ T cells, and IL-21 producing CD4^+^ T cells were significantly reduced by the 2-DG treatment (**Figures 5A** **–C**). Importantly, when HK2 was inhibited, the combined use of IGU did not further reduce the inhibitory effect (**Figures 5A** **–C**), indicating that IGU inhibited HK2 resulting in cTfh cell function inhibition.

### IGU Restrains cTfh Cell Function by Inhibiting Hif1α-HK2 Axis

Recent experiments have shown that mTOR and Hif1α perform important functions in glucose metabolism (26). In our study, we evaluated the expression of mTOR and Hif1α in RA and HC CD4^+^ T cells. We found that the level of p-mTOR in RA and HC CD4^+^ T cells was similar (**Figure S5A**). However, the expression of Hif1α in RA-CD4^+^ T cells was higher than in HC (**Figure S5B**).

**Figure 6:**
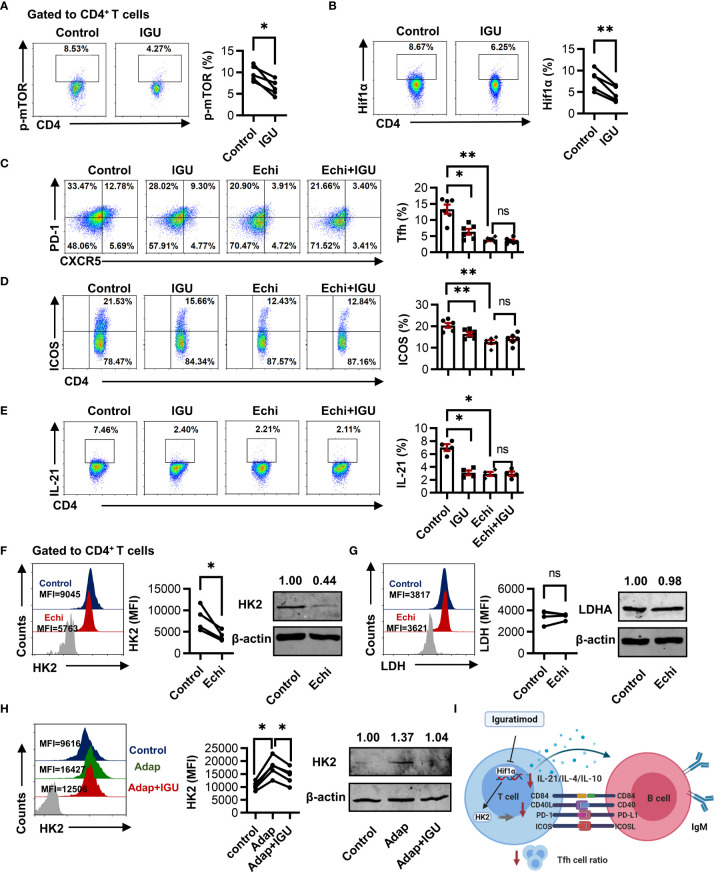
IGU restrains cTfh cell function by inhibiting the Hif1α-HK2 axis. **(A, B)** RA-PBMCs were activated by anti-CD3/CD28 antibody (2 μg/ml) and treated with or without IGU for 3 days, and the levels of p-mTOR **(A)** and Hif1α **(B)** were analyzed by flow cytometry (n = 5). **(C–E)** RA-PBMCs were pretreated with anti-CD3/CD28 antibody and Hif1α inhibitor Echinimycin (Echi) for 4 hours and then treated with IGU or DMSO for 3 days. The frequencies of Tfh cells (n = 6), ICOS^+^CD4^+^ T cells (n = 6), and IL-21 producing CD4^+^ T cells (n = 4) were determined by flow cytometry. **(F, G)** RA-PBMCs were treated with Echinomycin (Echi) for 24 hours, HK2 (n = 4) and LDH (n = 4) in RA-CD4^+^ T cells were measured. **(H)** RA-PBMCs were pretreated by anti-CD3/CD28 antibody and Adaptaquin (the activator of Hif1α) (5 μM), and then treated with IGU for 72 hours. The expression of HK2 (n = 5) was measured. **(I)** Schematic depicting the described findings: IGU restrains RA cTfh cell function by inhibiting the Hif1α-HK2 axis. MFI, mean fluorescence intensity. ns, no significance; *P < 0.05; **P<0.01.

We further dissected whether mTOR-Hif1α signaling was involved in IGU-mediated cTfh cells inhibition. We found that IGU treatment down-regulated both p-mTOR and Hif1α in RA-CD4^+^ T cells (**Figures 6A** **, B**). We then used the specific pharmacological inhibitors Rapamycin and Echinomycin against mTOR and Hif1α respectively to confirm the functional importance of mTOR-Hif1α signaling in RA-CD4^+^ T cells inhibition induced by IGU. We observed that Echinomycin strongly reduced the frequencies of cTfh cells, the expression of ICOS, and the IL-21 produced by CD4^+^ T cells (**Figures 6C** **–E**). Importantly, When Hif1α was inhibited, combined treatments with Echinomycin and IGU did not further reduce the inhibitory effect (**Figures 6C** **–E**). The frequencies of cTfh cells, ICOS, and IL-21 were also decreased by mTOR antagonist Rapamycin (**Figure S6**), but the frequency of cTfh cells, the activated molecules on CD4^+^ T cells, and the IL-21 production by CD4^+^ T cells were further decreased by adding IGU. We ensured that Echinomycin and Rapamycin did not affect cell apoptosis (**Figure S4**). These results indicate that Hif1α is critically involved in IGU mediated RA-cTfh cell suppression and IGU could decrease mTOR which might partially contribute to the reduction of cTfh cells. Moreover, we found that treatment with Hif1α inhibitor significantly decreased HK2 but not LDH expression (**Figures 6F** **, G**). In addition, we activated the Hif1α function with Adaptaquin. We found that activation of Hif1α signaling dramatically upregulated the expression of HK2 in RA-CD4^+^ T cells and IGU treatment reconstituted HK2 expression (**Figure 6H**). These results collectively suggest that IGU restrains RA cTfh cell function by inhibiting the Hif1α-HK2 axis (**Figure 6I**).

## Discussion

The current studies on IGU have certain limitations and the drug target of IGU has been controversial, which greatly limits a broader application of this drug. In this study, we demonstrate that IGU can directly restrain RA-cTfh cell function by inhibiting the Hif1α-HK2 axis. Collectively, our study uncovered a potential mechanism of IGU, which is to inhibit cTfh cell function by constraining glucose metabolism.

RA is a systemic autoimmune disease that is characterized by immune cell infiltration in the joint. The presence of autoantibodies is a hallmark for the disease, including rheumatoid factor and antibodies against post-translational modified proteins like citrullination (ACPA) and carbamylation (anti-CarP antibodies) (27). Antibody responses can be induced in a T cell-dependent or independent manner (22). Tfh cells play critical roles in the humoral immune response by supporting B cell proliferation and differentiation (28–30). A reduction of serum immunoglobulin concentration in RA patients following IGU treatment indicates that the regulation of humoral response is a key aspect of IGU (17, 31). Some evidence suggests that IGU reduces immunoglobulin production by acting on B cells without modifying B cell proliferation (16, 32). However, in our study, we observed that IGU could directly act on RA-CD4^+^ T cells. We confirmed that IGU inhibited T cell-dependent antibody production through a T-B cell co-culture experiment. Even if only significant changes in IgM were observed in our experiment, this may be due to the IgM antibody being first secreted at the initial stage of B cell activation. If the T-B co-culture time is prolonged, significant changes in IgG may be observed. We further found that IGU could reduce the percentages of cTfh cells and Tfh cell-related molecules and cytokines. These results show that IGU inhibits the function of RA-cTfh cells, indicating that Tfh cells may be a target of IGU in RA treatment.

A direct link between dysregulated glucose metabolism in T cell and autoimmunity have been reported (25, 33, 34). CD4^+^ T cells from lupus patients and lupus-prone mice display high demand for glucose and depend on the rapid production of ATP, requiring both mitochondrial activity and cytoplasmic glycolysis (35). On the contrary, RA T cells shift glucose into the pentose phosphate pathway (36, 37). Our current studies demonstrate that compared with HC-CD4^+^ T cells, the expression of GLUT1 and HK2 in RA-CD4^+^ T cells is increased, suggesting that RA-CD4^+^ T cells have more active glucose metabolism than HC-CD4^+^ T cells. The results suggest that targeting the glucose metabolism of CD4^+^ T cells may be a therapeutic strategy for RA. The inhibition of key metabolic pathways that are upregulated during T cell inflammation presents a credible therapeutic strategy for the treatment of autoimmune diseases (38). Of note, some chemical immunosuppressive drugs widely prescribed for the treatment of autoimmune and inflammatory diseases, also target T-cell metabolism and metabolic checkpoints (14). Interestingly, we found that the glucose uptake ability was significantly down-regulated in RA-CD4^+^ T cells after treatment with IGU. We also found that IGU could significantly inhibit the expression of HK2. Importantly, we confirmed that the inhibitory effect of IGU on RA-Tfh cells was dependent on the inhibition of HK2 by using specific pharmacological inhibitors 2-DG. Concurrently, we also found that the LDH expression and lactate production of CD4^+^ T cells were significantly decreased in the presence of IGU. Real-time bioenergetic profiles also showed that IGU treated CD4^+^ T cells have lower glycolysis and glycolytic capacity, which implied IGU also played a key role in regulating the T cells in other diseases which are characterized by glycolysis, such as systemic lupus erythematosus (SLE). More interestingly, we found that the application of 2-DG could significantly inhibit the expression of CXCR5, implying that glycolysis plays a key role in the function of CXCR5, which is worthy of further study.

Recent studies have demonstrated that mTOR signaling plays a critical role in the regulation of glucose uptake and energy balance (12). Hif1α also serves as a key transcription factor that performs important functions in the regulation of cellular metabolism, especially in the regulation of HK2 expression (13). Increased mTOR activity promoted Tfh cell responses, and Hif1α-dependent glycolysis is required for Tfh cells (39–41). Therefore, we hypothesized that mTOR-Hif1α signaling is involved in IGU-mediated RA cTfh cell inhibition. Strikingly, we found that IGU could down-regulated both p-mTOR and Hif1α. By using the specific pharmacological inhibitors Rapamycin and Echinomycin, we confirmed the functional importance of Hif1α in RA-Tfh cell suppression induced by IGU. However, IGU could also decrease mTOR which might partially contribute to the reduction in Tfh differentiation.

The limitations of this study include lacking animal experiments and clinical verification. While our studies provide evidence that IGU restrains RA-cTfh cell function by inhibiting the Hif1α-HK2 axis, this novel concept has not been validated in a natural RA microenvironment. The regulatory effect of IGU on T cell function and metabolism needs to be further verified in vivo in animal models. Furthermore, the clinical data on the function and glucose metabolism in CD4^+^ T cells from RA patients treated only with IGU are needed. *In vitro*, IGU reduces T cell proliferation which leads to lower numbers of total T cells including Tfh cells, which might explain *in vivo*: fewer Tfh numbers lead to less antibody production. In addition, the differentiation of Tfh cells affected by IGU may be the main reason for the altered proportion of Tfh. Further study is needed to elucidate the molecular mechanism of IGU affecting the differentiation of Tfh in future research. In the T-B coculture system, we used the same number of CD4^+^ T cells (treated or not with IGU), and we could see a defect in antibody production in IGU treated group. The same number of Tfh cells should be used for co-culture experiments to further confirm that IGU inhibits the function of Tfh cells. And we also found that Th1, Th17, and Tph cells were also significantly inhibited by IGU, and this mechanism needs to be further explored.

In conclusion, we clarified that IGU could restrain cTfh cell function by inhibiting glucose metabolism *via* Hif1α-HK2 axis in RA. This was the first study to demonstrate that IGU can inhibit CD4^+^ T cell function by regulating cell glucose metabolism. Importantly, our study demonstrates the potential application of IGU in the treatment of diseases related to abnormal metabolism and function of Tfh cells.

## Data Availability Statement

The original contributions presented in the study are included in the article/**Supplementary Material**. Further inquiries can be directed to the corresponding authors.

## Ethics Statement

The studies involving human participants were reviewed and approved by ethics committee of the Dalian Medical University. The patients/participants provided their written informed consent to participate in this study.

## Author Contributions

ZB performed the experiments and wrote the manuscript; ZB, ZL, and RL analyzed the data and constructed the figures; YT and XY provided technical assistance and contributed to writing the paper; MJ contributed to writing the paper; GW and XL designed the research and contributed to writing the paper. All authors contributed to the article and approved the submitted version.

## Funding

This work was supported by the National Natural Science Foundation of China [grant numbers 82071834, 81801609, 82101896]; the Liaoning Distinguished Professor program [Liao taught 2018 to 2020]; the “Seedling” project of young scientific and technological talents of Liaoning Provincial Department of Education [grant number LZ2019061]; China International Medical Foundation and Rheumatoid Special Fund [grant number Z-2018-40]; Science and Technology Innovation Foundation of Dalian (2021JJ13SN48); Dalian key laboratory of human microorganism homeostasis and immunological mechanism research of diseases.

## Conflict of Interest

The authors declare that the research was conducted in the absence of any commercial or financial relationships that could be construed as a potential conflict of interest.

## Publisher’s Note

All claims expressed in this article are solely those of the authors and do not necessarily represent those of their affiliated organizations, or those of the publisher, the editors and the reviewers. Any product that may be evaluated in this article, or claim that may be made by its manufacturer, is not guaranteed or endorsed by the publisher.
