# Supplementary material for: Iguratimod Restrains Circulating Follicular Helper T Cell Function by Inhibiting Glucose Metabolism via Hif1α-HK2 Axis in Rheumatoid Arthritis
Source: Front Immunol. 2022 Jun 1;13:757616. doi: 10.3389/fimmu.2022.757616 (PMC9199372; doi:10.3389/fimmu.2022.757616)
Supplement: Supplementary file 1 [file DataSheet_1.docx]

Supplementary Material

## Supplementary Tables

##### Supplemental Table 1. Antibodies used for Flow cytometry

| Antibodies | Source | Clone number |
| --- | --- | --- |
| Anti-HK2 Alexa Fluor 647 | Abcam | EPR20839 |
| Anti-LDH PE | Abcam | EP1563Y |
| Anti-Human CD4 FITC | eBioscience | RPA-T4 |
| Anti-Human ICOS PE | eBioscience | ISA-3 |
| Anti-Human CXCR5 APC | eBioscience | MU5UBEE |
| Bcl-6 Monoclonal Antibody | eBioscience | BCL-UP |
| Anti-Human IL-17A PE | eBioscience | eBio64DEC17 |
| Anti-Human IFN-γ APC | eBioscience | 4S.B3 |
| Anti-Human IL-4 PE | eBioscience | 8D4-8 |
| Anti-Human CD84 APC | eBioscience | 2G7 |
| Anti-Human CD25 APC | eBioscience | BG96 |
| Anti-Human p-mTOR PE | eBioscience | MRRBY |
| Anti-Human CD138 PE | eBioscience | Syndecan-1 |
| Anti-Human PD-1 FITC | Biolegend | EH12.2H7 |
| Anti-Human CD4 PerCP-Cy5.5 | Biolegend | RPA-T4 |
| Anti-Human CD69 PE | Biolegend | FN50 |
| Anti-Human Hif-1α PE | Biolegend | 546-16 |
| Anti-Human CD40L PE/Cyanine7 | Biolegend | 24-31 |
| Anti-Human PD-1 PE/Cyanine7 | Biolegend | EH12.2H7 |
| Anti-Human GLUT1 PE | RD | FAB1418P |
| Anti-Human CD4 APC | BD | RPA-T4 |
| Anti-Human CD19 APC | BD | SJ25C1 |
| Anti-Human IL-21 PE | BD | 3A3-N2.1 |
| Anti-Human IL-10 APC | BD | JES3-19F1 |
| Fixable Viability Dye eFluor 780 | eBioscience | - |

##### Supplemental Table 2. Primers used for quantitative PCR

| Genes | Primers |
| --- | --- |
| β-actin Foward | CATGTACGTTGCTATCCAGGC |
| β-actin Reverse | CTCCTTAATGTCACGCACGAT |
| HK2 Foward | TTGACCAGGAGATTGACATGGG |
| HK2 Reverse | CAACCGCATCAGGACCTCA |
| PFKM Foward | AATCTGCAAGAAAGCAGCGG |
| PFKM Reverse | GCAGCATTCATACCTTGGGC |
| PKM1/2 Foward | ATGTCGAAGCCCCATAGTGAA |
| PKM1/2 Reverse | TGGGTGGTGAATCAATGTCCA |
| LDHA Foward | TTGACCTACGTGGCTTGGAAG |
| LDHA Reverse | GGTAACGGAATCGGGCTGAAT |
| PFKFB3 Foward | CTCGCATCAACAGCTTTGAGG |
| PFKFB3 Reverse | TCAGTGTTTCCTGGAGGAGTC |
| G6PD Foward | GGCCGTCACCAAGAACATTC |
| G6PD Reverse | TGGTCGATGCGGTAGATCTG |
| BATF Foward | TGGCAAACAGGACTCATCTG |
| BATF Reverse | CTGTTTCTCCAGGTCTTCGC |
| Bcl-6 Foward | GTTTCCGGCACCTTCAGACT |
| Bcl-6 Reverse | CTGGCTTTTGTGACGGAAAT |

## Supplementary Figures


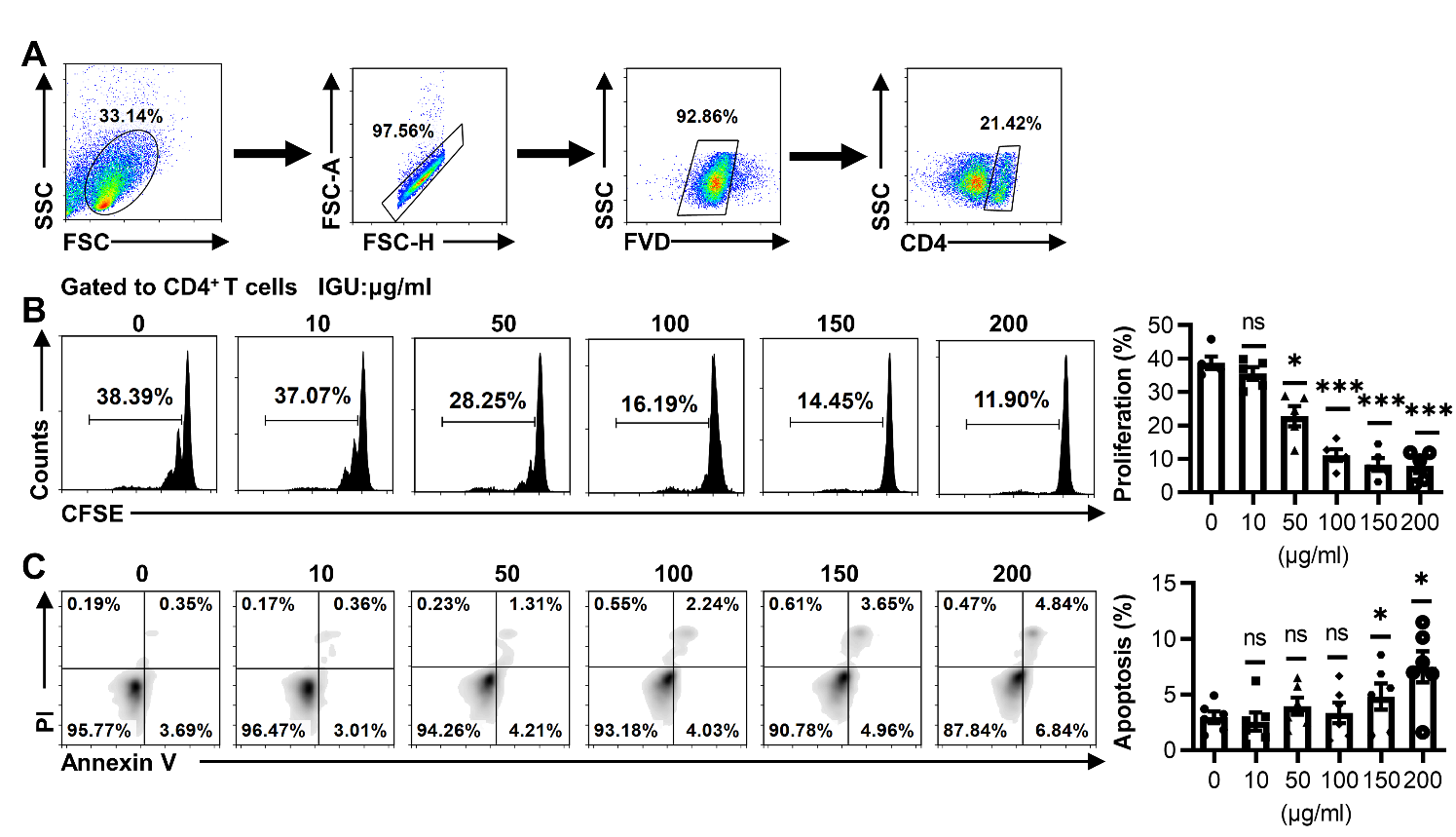


##### S1. Drug concentration screening. The PBMCs from healthy donors activated by anti-CD3/CD28 antibody (2 μg/ml) were added with different concentrations of IGU (0, 10, 50, 100, 150, 200 μg/ml) for 72 hours. (A) Gating strategy of CD4^+^ T cells. (B) The proliferation of CD4^+^ T cells was detected by the CFSE labeling method (n = 5). (C) The apoptosis of CD4^+^ T cells was determined by flow cytometry after treatment with different concentrations of IGU for 24 hours (n = 6). The data of early apoptotic cells (AV^+^PI^−^) were shown. Symbols represent individual subjects. ns, no significance; *, *P* < 0.05; ***, *P* < 0.001.


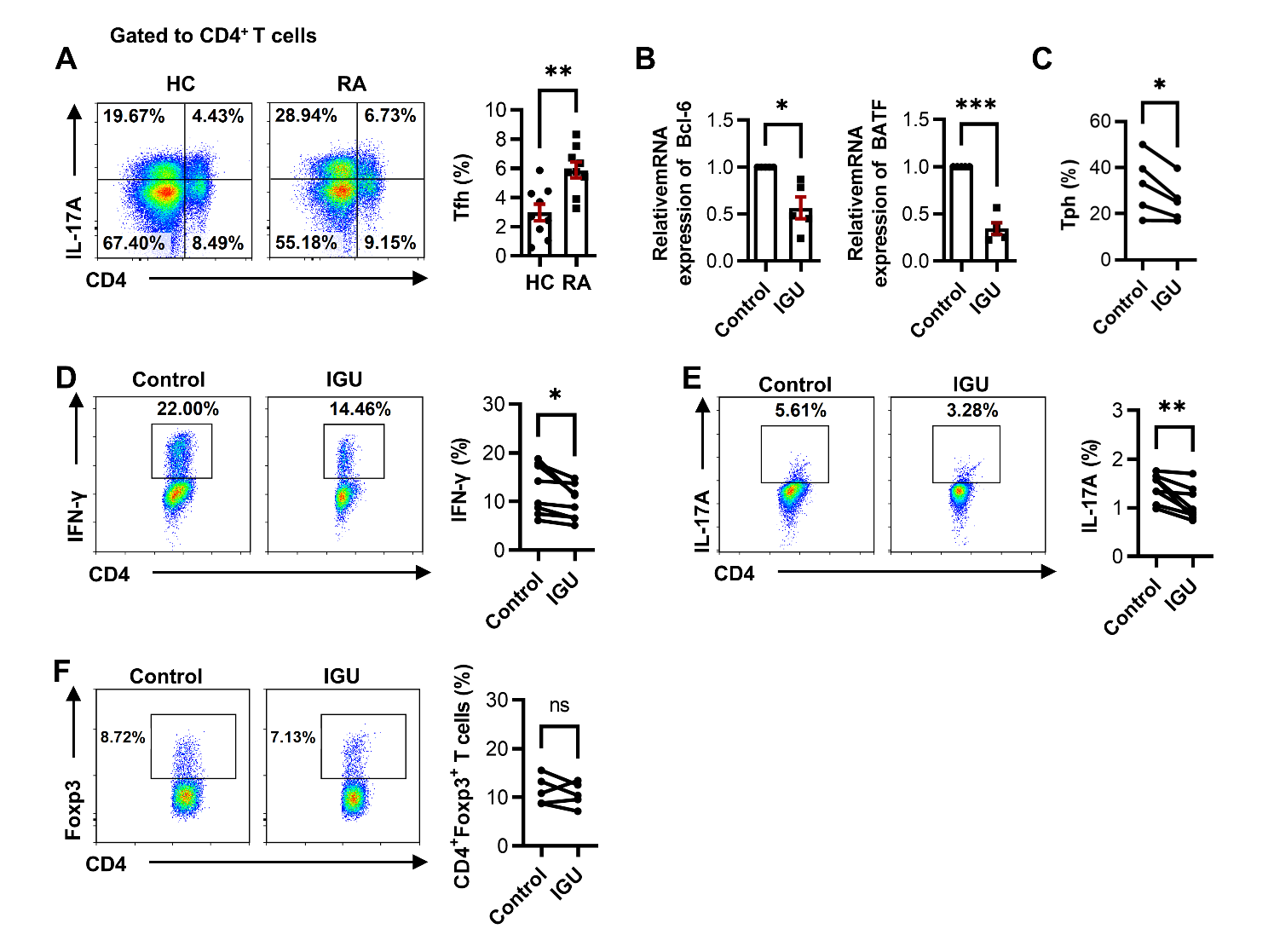


**S2. Effect of IGU on T cell subsets.** (A) The difference in circulating Tfh cells between RA patients (n = 9) and HC (n = 9). (B) Purified RA-CD4^+^ T cells were cultured in the presence of IGU or DMSO for 24 hours, and relative mRNA expression levels of Bcl-6 and BATF were determined by qPCR. (C-F) RA-PBMCs were cultured in the presence or absence of IGU for 72 hours. The percentage of Tph (CD4^+^PD-1^+^CXCR5^-^) (n = 5), Th1 (CD4^+^IFN-γ^+^) (n = 8), Th17 (CD4^+^IL-17A^+^) (n = 8) and Treg (CD4^+^Foxp3^+^) (n = 5) were detected by flow cytometry. Symbols represent individual subjects. ns, no significance; *, *P* < 0.05; **, *P* < 0.01.


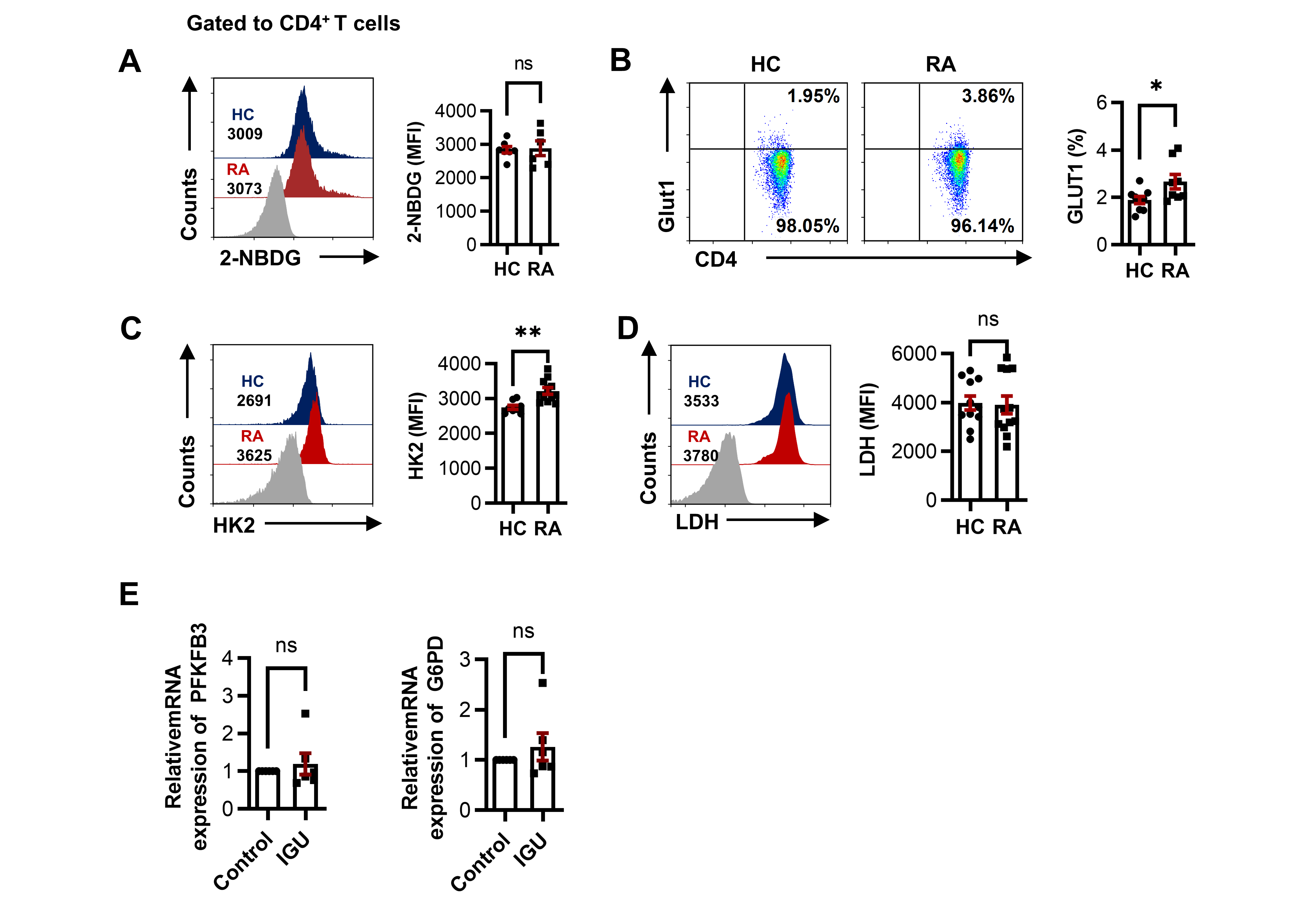


##### S3. RA-CD4^+^ T cells have more active glucose metabolism than HC-CD4^+^ T cells. (A) PBMCs from RA patients and HC were labeled with 2-NBDG for 20 min to determine the glucose uptake of CD4^+^ T cells. (B-D) The expression of GLUT1 (B), HK2 (C), and LDH (D) in RA and HC CD4^+^ T cells were detected by flow cytometry. (E) Purified RA-CD4^+^ T cells were cultured in the presence of IGU or DMSO for 24 hours, and relative mRNA expression levels of PFKFB3 and G6PD were determined by qPCR. Symbols represent individual subjects. MFI: mean fluorescence intensity. ns, no significance; *, *P* < 0.05; **, *P* < 0.01.

**
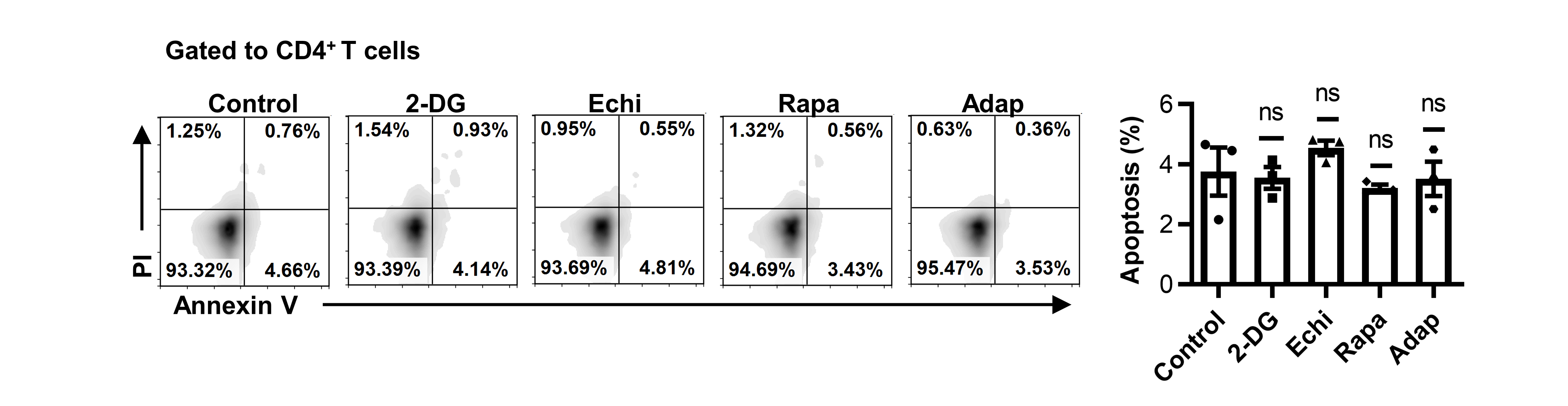
**

##### S4. The effects of inhibitors and stimulants on CD4^+^ T cell apoptosis. The apoptosis of CD4^+^ T cells was determined by flow cytometry after 2-DG treatment, Echinomycin, Rapamycin, and Adaptaquin for 24 hours (n = 3). The data of early apoptotic cells (AV^+^PI^−^) were shown. Symbols represent individual subjects. ns, no significance.


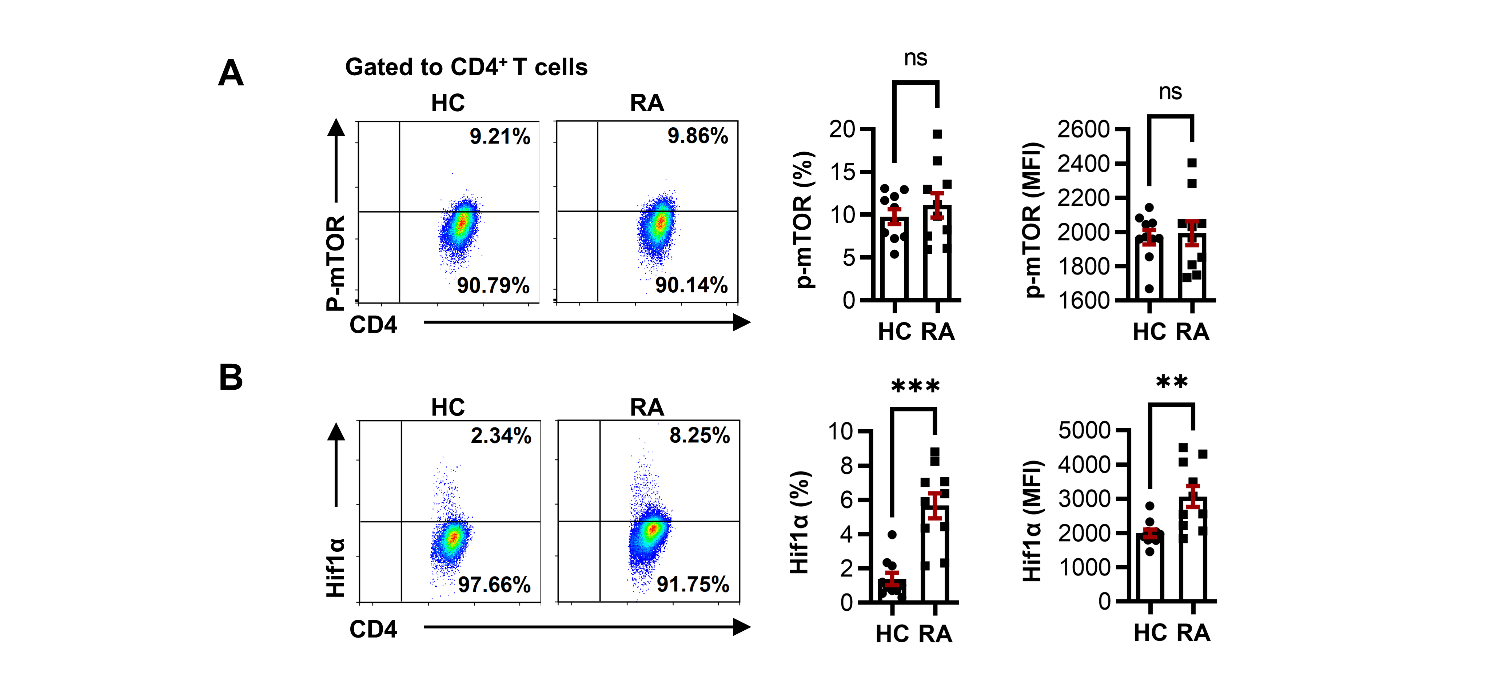


##### S5. The expression of p-mTOR and Hif1α in RA and HC CD4^+^ T cells. The expression of p-mTOR (A) and Hif1α (B) between RA (n = 10) and HC (n = 10) CD4^+^ T cells were detected by flow cytometry. Symbols represent individual subjects. MFI: mean fluorescence intensity. ns, no significance; **, *P* < 0.01; ***, *P* < 0.001.


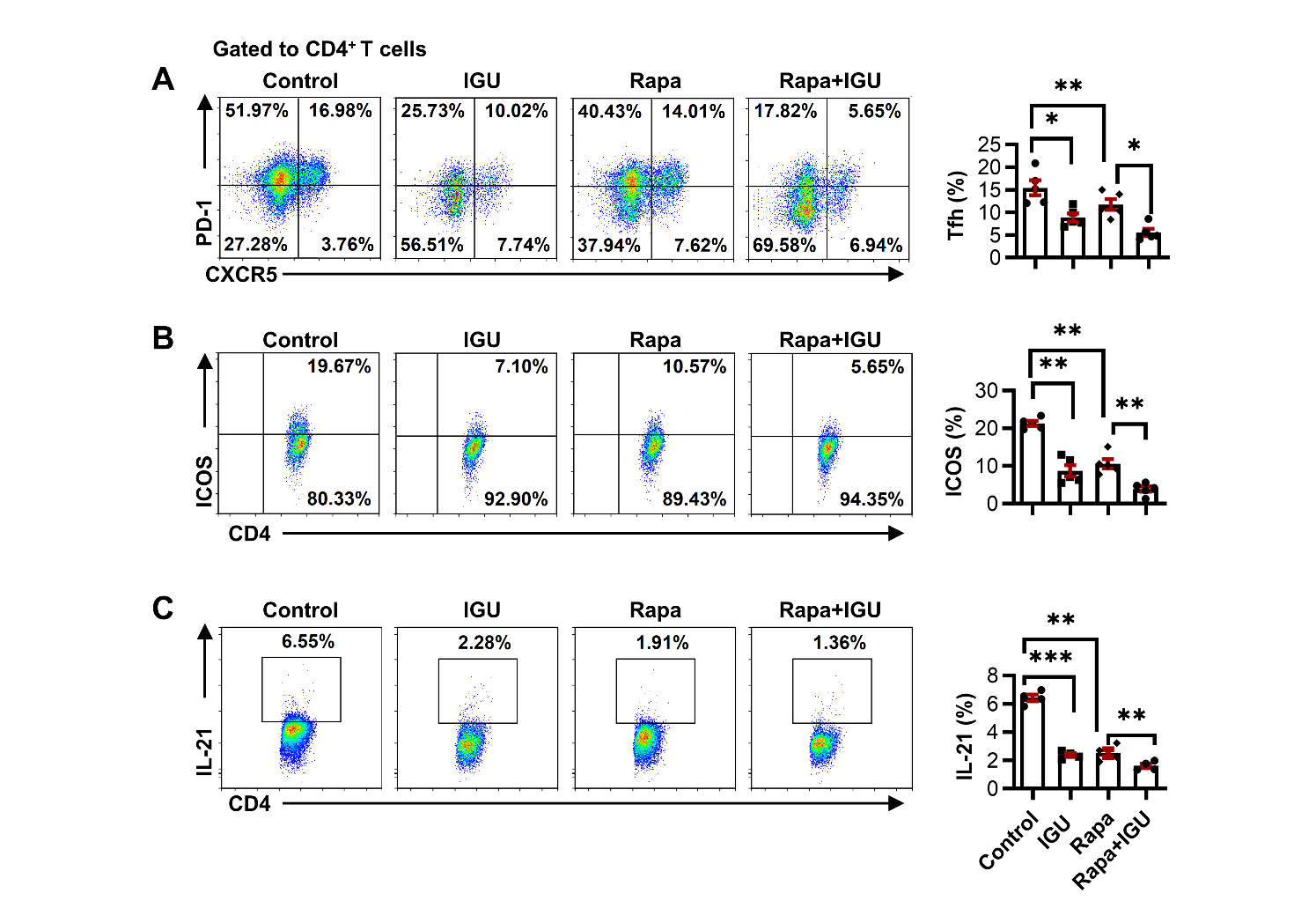


##### S6. The inhibition of function in Tfh cells by IGU is independent of mTOR. RA PBMCs were activated by anti-CD3/CD28 antibody (2 μg/ml) and pretreated with mTOR inhibitor Rapamycin (Rapa) for 4 hours, and then treated with IGU or DMSO for 3 days. (A) The frequencies of Tfh cells (n = 5), (B) CD4^+^ICOS^+^ T cells (n = 5), and (C) IL-21 producing CD4^+^ T cells (n = 4) were determined by flow cytometry. *, *P* < 0.05; **, *P*<0.01; ***, *P* < 0.001.
